# Nursing Teleconsultation for the Outpatient Management of Patients with Cardiovascular Disease during COVID-19 Pandemic

**DOI:** 10.3390/ijerph18042087

**Published:** 2021-02-21

**Authors:** Vincenzo Russo, Roberta Cassini, Valentina Caso, Chiara Donno, Annunziata Laezza, Maria Naddei, Alfonso Fiorelli, Paolo Golino, Gerardo Nigro

**Affiliations:** 1 Cardiology Unit, Department of Medical Translational Science, University of Campania “Luigi Vanvitelli” , Monaldi Hospital, 80131 Naples, Italy; robertacassini8@gmail.com (R.C.); paolo.golino@unicampania.it (P.G.); gerardo.nigro@unicampania.it (G.N.); 2 Cardiac Diagnostic Unit, Monaldi Hospital, 80131 Naples, Italy; valepica8@yahoo.it (V.C.); chiara.donno@ospedalideicolli.it (C.D.); nunzialaezza@libero.it (A.L.); 3 Day Hospital Surgery Unit, University of Campania “Luigi Vanvitelli”, 80131 Naples, Italy; maria.naddei@unicampania.it; 4 Thoracic Surgery Unit, University of Campania “Luigi Vanvitelli”, 80131 Naples, Italy; alfonso.fiorelli@unicampania.it

**Keywords:** COVID-19, telemedicine, nursing teleconsultation, cardiovascular risk, dyslipidemia, atrial fibrillation, outbreak

## Abstract

**Introduction:** During the COVID-19 outbreak, non-urgent clinic visits or cardiac interventional procedures were postponed to a later date, and the implementation of telemedicine has guaranteed continuity of care for patients with chronic diseases. The aim of our study was to describe the medical interventions following nursing teleconsultation for the outpatient management of patients with cardiovascular diseases during the COVID-19 pandemic. **Materials and Methods:** All patients who did not attend the follow-up visit from 4 to 15 April 2020 at our institution and who were re-scheduled due to the COVID-19 lockdown were selected to be enrolled in the study. Each patient was followed by a semi-structured telephonic interview performed by a nurse. The outcomes of our study were to assess the patients’ adherence to nursing teleconsultation and the usefulness of nursing teleconsultation to detect clinical conditions in need of medical intervention. **Results:** In total, 203 patients (81%) underwent nursing teleconsultation in a mean time of 7 ± 3 days from the outpatient visit lost due to the COVID-19 lockdown. Furthermore, 53 patients (26%) showed poor adherence to nursing teleconsultation. Among the 150 patients (mean age 67 ± 10 years; 68% male) who completed the telephonic interview, the nursing teleconsultation revealed the need of medical intervention in 69 patients (46%), who were more likely at very high cardiovascular risk (77% vs. 48%; *p* < 0.0003) and who showed a higher prevalence of dyslipidemia (97% vs. 64%; *p* < 0.0001) and coronary artery disease (75% vs. 48%, *p* < 0.0008) compared to those not in need of any intervention. The up-titration of the lipid-lowering drugs (*n*: 32, 74%) was the most frequent medical intervention following the nursing teleconsultation. The mean time between the nursing teleconsultation and the date of the rescheduled in-person follow-up visit was 164 ± 36 days. **Conclusions:** Nursing teleconsultation is a simple and well-tolerated strategy that ensures the continuity of care and outpatient management for patients with cardiovascular diseases during the COVID-19 pandemic.

## 1. Introduction

The coronavirus disease 2019 (COVID-19) outbreak is a global health emergency related to a highly pathogenic human coronavirus responsible for the severe acute respiratory syndrome (SARS-CoV-2) [1]. The clinical course of the disease may be complicated by the onset of severe respiratory distress syndrome and/or multi-organ failure that may require hospitalization [2]; however, many infected patients remain asymptomatic or paucisymptomatic and are managed in outpatient settings [3]. Italy is among the countries most severely hit by COVID-19; on 1 January 2021 there were 2.129.376 total positive cases and 74.621 deaths [4]. Following the COVID-19 outbreak, from 10 March to 4 May 2020, the Italian government, in an attempt to contain the virus diffusion, adopted strict rules characterized by lockdown and social distancing [5]. During the COVID-19 outbreak and consequent lockdown period, non-urgent clinic visits or cardiac interventional procedures were postponed to a later date, and some changes in the patterns of hospital admissions have been observed [6,7,8]. International guidelines recommend that all high-risk individuals, including those with traditional cardiovascular risk factors and/or established atherosclerotic cardiovascular disease (ASCVD), stay at home if possible, primarily to limit potential exposure; moreover, clinicians and patients are strongly recommended to apply telehealth tools as an appropriate option to prevent and contain COVID-19 infection [9]. Early experience showed that the implementation and integration of telemedicine during the pandemic has guaranteed continuity of care for patients with chronic degenerative non-communicable diseases [10,11], and there was no experience concerning nurse involvement in telehealthcare delivery in Italy. Our study aimed to describe the medical interventions following nursing teleconsultation for the outpatient management of patients with cardiovascular diseases during the COVID-19 pandemic.

## 2. Materials and Methods

### 2.1. Study Design and Population

From the hospital healthcare database, which includes data from the electronic patient records of all patients followed at the cardiac diagnostic unit of the University of Campania “Luigi Vanvitelli”, all patients who missed the follow-up visit from 4 to 15 April 2020 at our institution and who were re-scheduled due to the COVID-19 lockdown were retrospectively selected to be enrolled in the study. Patients with a missing or untraceable telephone number or who did not give informed consent were excluded.

### 2.2. Nursing Teleconsultation

Each patient was followed by a semi-structured telephonic interview performed by a nurse. Patients’ demographics, cardiovascular risk factors, comorbidities, height, weight, blood pressure, body temperature, heart rate, and pharmacological treatments were collected; moreover, each patient was asked to send by email or dedicated mobile number the blood laboratory examinations that they should have exhibited during the missed follow-up visit.

### 2.3. Outcomes

The outcomes of our study were to assess the patients’ adherence to nursing teleconsultation and the usefulness of nursing teleconsultation to detect clinical conditions in need of medical intervention. The patient’s adherence to nursing teleconsultation was measured by the number of patients who completed the telephonic interview and sent the required blood laboratory examinations. The usefulness of nursing teleconsultation was assessed by calculating the number of medical interventions carried out. Moreover, the time between the missed in-person visit, nursing teleconsultation, and rescheduled in-person visit was calculated.

### 2.4. Statistical Analysis

Kolmogorov–Smirnov and Shapiro–Wilk tests were used to evaluate the distribution of continuous data. Normally distributed variables were expressed as the mean ± standard deviation (SD), whereas non-normal distributed ones as the median and interquartile range (IQR). Categorical variables were reported as numbers and percentages. Continuous normally distributed variables were compared by using the Student t-test; differences between non-normally distributed variables were tested with the Mann–Whitney U test. Categorical variables were compared with the chi-squared test, or Fisher exact test, when appropriate. For all tests, a *p*-value < 0.05 was considered statistically significant. Analyses were performed by using R version 3.5.1 (R Foundation for Statistical Computing, Vienna, Austria).

### 2.5. Ethics Approval

The study was conducted in accordance with the Declaration of Helsinki and was approved by the Local Ethical Committee (ID-210520). Written informed consent was obtained from all study participants before the commencement of data collection.

## 3. Results

**Figure 1:**
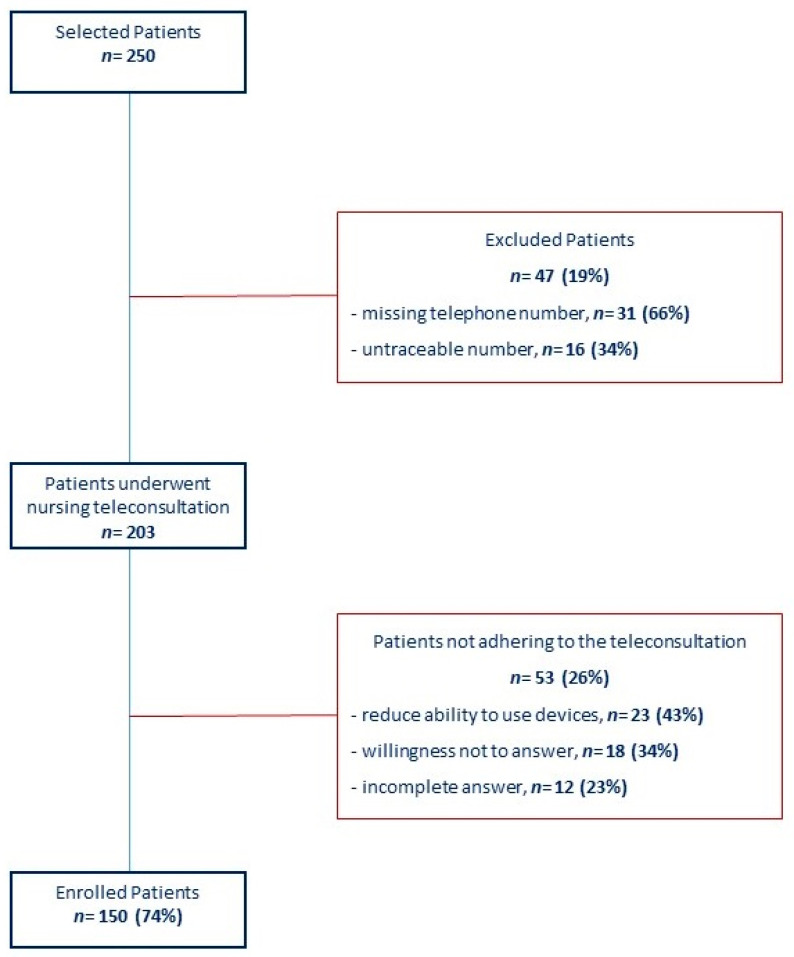
Inclusion graph of the study population.

As illustrated in the inclusion graph (Figure 1), we selected 250 patients who missed the follow-up visit from 4 to 15 April 2020 at our institution and who were re-scheduled due to the COVID-19 lockdown. In total, 47 patients (19%) were excluded for missing (*n*: 31; 66%) or untraceable phone number (*n*: 16; 34%); 203 patients (81%) underwent nursing teleconsultation in a mean time of 7 ± 3 days from the outpatient visit lost due to the COVID-19 lockdown. Furthermore, 53 patients (26%) showed poor adherence to nursing teleconsultation, mainly due to the reduced participant ability to use technological devices (*n*: 23; 43%), the unwillingness to answer (*n*: 18; 34%), and the incomplete answers to the structural interview (*n*: 12; 23%). Finally, 150 patients (mean age 67± 10 years; 68% male) who completed the nursing teleconsultation were analyzed. Baseline characteristics of the study population are summarized in Table 1.

**Figure 2:**
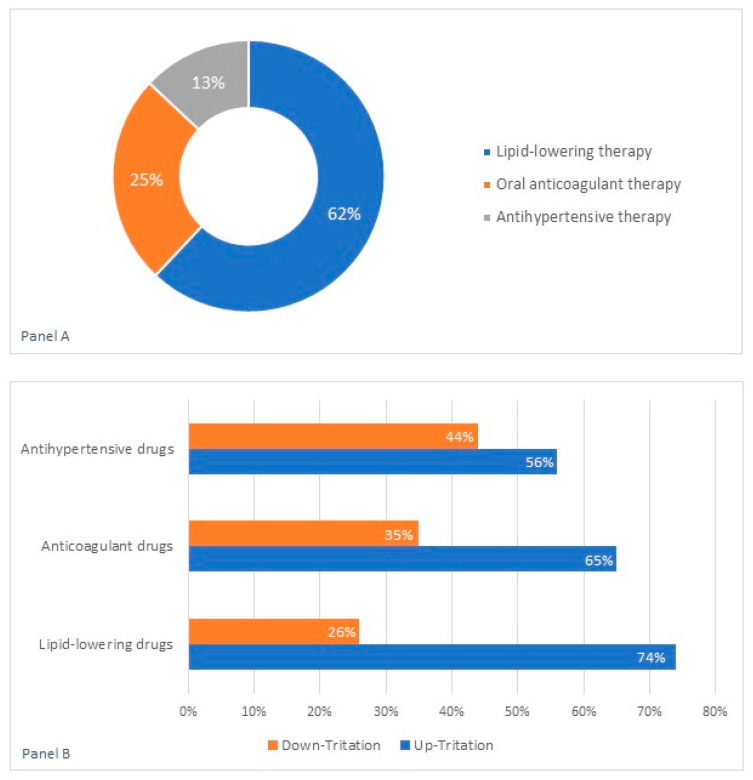
Type of therapy (Panel A) and dose adjustment (Panel B) following the nursing teleconsultation.

**Figure 3:**
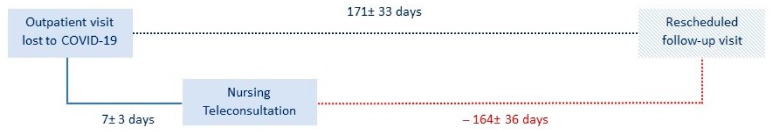
Time between the outpatient visit lost due to the COVID-19 lockdown, nursing teleconsultation and rescheduled in-person follow-up visit.

Hypertension was the most prevalent comorbidity (85%), followed by dyslipidemia (79%) and coronary artery disease (60%). Atrial fibrillation (22%), diabetes mellitus (22%), chronic obstructive pulmonary disease (23%), valvular heart disease (24%), and obesity (16%) were present in about one quarter of the study population. Based on these characteristics, 61% of the study population was considered at very high risk of cardiovascular events [12]. The nursing teleconsultation revealed the need for medical intervention in 69 patients (46%), who were more likely at very high cardiovascular risk (77% vs. 48%; *p* < 0.0003) and who showed a higher prevalence of dyslipidemia (97% vs. 64%; *p* < 0.0001) and coronary artery disease (75% vs. 48%, *p* < 0.0008) compared to those not in need of any intervention. The medical intervention was the optimization of the pharmacological treatments, including lipid-lowering (*n*: 43; 62%), anticoagulant (*n*: 17;25%), and antihypertensive (*n*: 9; 13%) therapy. The up-titration of the lipid-lowering drugs (*n*: 32, 74%) was the most frequent medical intervention following the nursing teleconsultation (Figure 2). The mean time between the outpatient visit lost due to the COVID-19 lockdown and the rescheduled in-person follow-up visit was 171± 33 days. The mean time between the nursing teleconsultation and the date of the rescheduled in-person follow-up visit was 164 ± 36 days (Figure 3).

## 4. Discussion

In Italy, telemedicine is a method of providing healthcare services through the use of innovative technologies in situations where the health professional and the patient are not in the same location. It involves the secure transmission of medical information and data in the form of texts, sounds, images, or other forms necessary for the prevention, diagnosis, treatment and subsequent monitoring of patients. Telemedicine services should be assimilated to any diagnostic/therapeutic health service; however, performance using telemedicine does not replace traditional healthcare provision in the doctor–patient personal relationship but integrates it to improve virtually efficacy, efficiency, and appropriateness [13]. There are three distinct ways to deliver telemedicine services: synchronous, asynchronous, and remote monitoring. Synchronous refers to the delivery of health information in real-time, through the use of digital devices, allowing for a live discussion with the patient or provider to deliver medical expertise. Asynchronous telemedicine refers to the “store-and-forward” technique, whereas a patient or physician collects medical history, images, and pathology reports and then sends it to a specialist physician for diagnostic and treatment expertise. The remote patient monitoring mode is achieved through an electronic device that records a continuous flow of information in real-time about any clinical patient’s change and transmits the data to a centralized website, which can be accessed in security the healthcare staff [14]. Teleconsultation is defined as synchronous or asynchronous consultation using information and communication technology to omit geographical and functional distance [15].

During the COVID-19 pandemic, a rapid implementation of telemedicine has also been shown in countries, like Italy, without integrated telemedicine within their national health care system [16]. The telemedicine helped the physicians to avoid direct physical contact and minimize the risk of SARS-CoV-2 transmission, lowering the morbidity and mortality rate for Covid-19, and finally provide continuous care to the community [9].

The strategy of replacing in-hospital visits with telecardiology has been effective in the short-term management of patients with cardiovascular disease, and patients would prefer to continue with remote monitoring compared to usual-care [17].

Nurse involvement in telemedicine has been suggested for establishing a first virtual approach to collect anamnestic data or educate the patient in the detection of vital parameters and collect laboratory and instrumental examinations carried out [10]; no experience about nursing teleconsultation for the management of patients with cardiovascular disease during the COVID-19 pandemic has been reported.

The main findings of the present study can be summarized as follows: the nursing teleconsultation based on a semi-structured phone interview and electronic transmission of medical documents was a well-tolerated tool, being accepted by 74% of the study population, for the management of patients with cardiovascular diseases during the COVID-19 pandemic. The lack of adherence to the nursing teleconsultation occurred in about a quarter of cases (26%), which was due to the reduced participant ability to use technological devices and the unwillingness to participate in the phone interview.

In the future, to expand the use of the nurse-based care delivery model through teleconsultation and to increase its credibility among patients, it would be necessary to officially recognize the telemedicine service and to address the evolving concerns related to reimbursement policies and licensing laws. Supportive training for nurse interviewers, oriented to ensure effective and clear communication, standardized telephone follow-up procedure, and successful data collection, should be provided. A national program for the digital literacy of elderly people should be activated to increase adherence to nursing teleconsultation. All these actions are mandatory to increase the use of telemedicine by physicians and mitigating the disruptions of care and enhancing the patients’ health during the COVID-19 pandemic.

In our study population, the nursing teleconsultation revealed the need to optimize the pharmacological therapy in about the half of study population; in particular, the up-titration of lipid-lowering and the adjustment of oral anticoagulant dose were the most frequently undertaken medical actions. This evidence supported the need for the continuity of care and outpatient management for patients with and at high risk for cardiovascular disease during the COVID-19 pandemic. Based on our results, 62% of the study population did not achieve the therapeutic target of LDL cholesterol in relation to their cardiovascular risk profile; in particular, in 74% of cases, it was necessary to increase the titration dose of the lipid-lowering drug, confirming the suboptimal LDL control among European patients at high risk of CVD [18]. The cardiovascular residual risk is defined as the residual risk of incident vascular events or progression of established vascular damage persisting in patients treated with current evidence-based recommended care, including the risk that was established from risk factors, such as dyslipidemia, high blood pressure, and the risk related to emerging or newer risk factors [19]. The global approach to cardiovascular risk should be focused on lifestyle optimization (smoking cessation, diet, exercise, and weight loss), LDL-c lowering therapy (statins, ezetimibe, or PCSK9-inhibitors), and treatment of atherogenic dyslipidemia with additional therapy targeting TG/HDL-c abnormalities [12]. Considering the worrying reduction in admissions for acute myocardial infarction and the parallel increase in case fatality and complication rates observed across Italy [8], the careful management of cardiovascular residual risk is of pivotal importance for the overall population during the COVID-19 pandemic. Cardiovascular prevention requires modern preventive cardiology programs delivered by interdisciplinary teams of healthcare professionals addressing all aspects of lifestyle and risk factor management to reduce the risk of recurrent cardiovascular events [20].

In 25% of our study population, the nursing teleconsultation revealed the need to adjust the oral anticoagulant dose; in the majority of cases (65%), an inadequately low dose of NOAC was found. The association between DOACs and inappropriate low dosing has been explained by the physicians’ fear of bleeding events or by the therapeutic inertia in the follow-up. NOAC underdosing has been associated with an increased risk of thromboembolic events [21]; consequently, it is of pivotal importance to prescribe an appropriate dosage, based on the summary of product characteristics (SmPC), in order to obtain in a real-world setting the same benefits demonstrated in randomized clinical trials [22]. In the remaining cases (35%), the nursing teleconsultation revealed the need for a reduction in the standard dose due to a worsening of renal function. Although the impact on renal function is lower for NOACs than for VKAs, the evaluation of creatinine and glomerular filtration rate according to Cockcroft-Gault should be performed for the early detection of a common cause of dose reduction [23].

**Figure 4:**
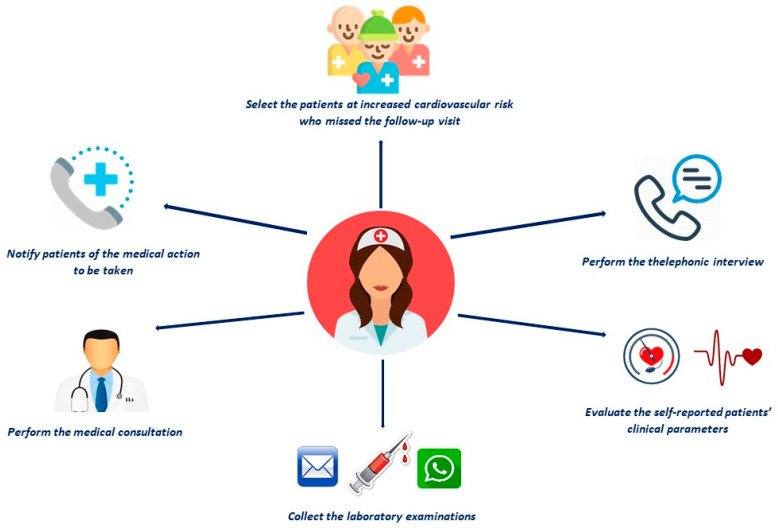
The nursing-based teleconsultation model.

These data confirm that the pre-specified follow-up schedule for patients in anticoagulant therapy should not be lost during the COVID-19 pandemic, in particular for frail, elderly people with high cardiovascular risk and prevalent comorbidities, such as dyslipidemia and ischemic heart disease. Although NOACs are safer than VKAs in some different clinical settings [23,24,25,26,27,28,29,30], an inappropriate dose may predispose to both hemorrhagic and thrombotic events; moreover, the use of any anticoagulant is associated with some drug–drug interactions, which may increase the risk of serious bleeding or diminish stroke protection. The teleconsultation should be oriented to evaluate the blood sampling (including hemoglobin, renal, and liver function); to check the adherence; and to re-assess if the chosen NOAC or its dose is the best for the patient, according to age, weight, or renal function [31,32,33]. Figure 4 shows the nursing-based teleconsultation model applied at our institution during the COVID-19 pandemic.

In our experience, the nursing teleconsultation significantly anticipated the optimization of the pharmacological treatment by about 4 months compared to the rescheduled in-person follow-up visit. Considering that the benefit of lowering LDL cholesterol depends on both the timing and the magnitude of LDL reduction, the strategy to implementing successful early intervention may improve the health of the population, and undoubtedly provide socioeconomic benefit by avoiding the costly complications of ASCVD [34].

## 5. Conclusions

Nursing teleconsultation is a simple and well-tolerated strategy that ensures the continuity of care and outpatient management for patients with cardiovascular diseases during the COVID-19 pandemic; however, since about a quarter of enrolled patients did not adhere to teleconsultation, the education and training of both nurses and physicians should be considered a goal of healthcare services. Based on our data, from the perspective of resource management during the pandemic phase, teleconsultation services should prioritize patients at very high cardiovascular risk or who are taking oral anticoagulants in order to optimize pharmacological therapies.

## Figures and Tables

**Table 1:** Clinical characteristics of the study population (*n*: 150) according to the need (intervention group) or not (target group) of medical intervention following nursing teleconsultation.

Variables	Overall Population *n*: 150	Intervention Group *n*: 69	Target Group *n*: 81	*p* Value
Age (mean ± SD)	67 ± 10	66 ± 3	67 ± 10.4	0.89
Male. *n* (%)	102 (68)	47 (68)	55 (68)	0.99
Female. *n* (%)	48 (32)	63 (91)	65 (80)	0.06
Smokers. *n* (%)	23 (15)	12 (17)	11 (14)	0.5
Hypertension. *n* (%)	128 (85)	15 (22)	10 (12)	0.13
Obesity. *n* (%)	25 (17)	67 (97)	52 (64)	0.0001
Dyslipidemia. *n* (%)	119 (79)	18 (26)	17 (21)	0.5
Diabetes mellitus. *n* (%)	33 (22)	52 (75)	39 (48)	0.0008
CAD. *n* (%)	91(61)	17(25)	16 (20)	0.5
Very high CV risk. *n* (%)	92 (61)	53(77)	39 (48)	0.0003
COPD. *n* (%)	35 (23)	14 (20)	20 (25)	0.51
AF. *n* (%)	34 (23)	3.18 (1.41)	3.04 (1.52)	0.77
CHA_2_DS_2_VASc (mean ± SD)	3.10 (1.5)	2.3 (0.1)	2 (0.96)	0.28
HASBLED (mean ± SD)	2.05 (1.18)	19 (27)	18 (22)	0.51
Valvular Heart Diseases. *n* (%)	37 (25)	58 (84)	65 (80)	0.57
Lipid-lowering therapy. *n* (%)	123(82)	32 (46)	19 (23)	0.10
Anticoagulant therapy. *n* (%)	51 (34)	38 (55)	41 (51)	0.72
Antihypertensive therapy. *n* (%)	79 (52)	38 (55)	41 (51)	0.72

CAD: coronary artery disease; CV: cardiovascular; COPD: chronic obstructive pulmonary disease; AF: atrial fibrillation.

## Data Availability

The data presented in this study are available on request from the corresponding author.
